# The molecular and cellular signatures of the mouse eminentia thalami support its role as a signalling centre in the developing forebrain

**DOI:** 10.1007/s00429-015-1127-3

**Published:** 2015-10-12

**Authors:** Kevin Kofi Adutwum-Ofosu, Dario Magnani, Thomas Theil, David J. Price, Vassiliki Fotaki

**Affiliations:** 1 The University of Edinburgh, Centre for Integrative Physiology, Hugh Robson Building, George Square, Edinburgh, EH8 9XD UK; 2 Department of Anatomy, College of Health Sciences, University of Ghana, Accra, Ghana; 3 MRC Centre for Regenerative Medicine, University of Edinburgh, Edinburgh, EH16 4SB UK

**Keywords:** Mouse, Development, Eminentia thalami, Signalling centre, Wnt/β-catenin signalling

## Abstract

The mammalian eminentia thalami (EmT) (or thalamic eminence) is an embryonic forebrain structure of unknown function. Here, we examined the molecular and cellular properties of the mouse EmT. We first studied mRNA expression of signalling molecules and found that the EmT is a structure, rich in expression of secreted factors, with *Wnts* being the most abundantly detected. We then examined whether EmT tissue could induce cell fate changes when grafted ectopically. For this, we transplanted EmT tissue from a tau-GFP mouse to the ventral telencephalon of a wild type host, a telencephalic region where Wnt signalling is not normally active but which we showed in culture experiments is competent to respond to Wnts. We observed that the EmT was able to induce in adjacent ventral telencephalic cells ectopic expression of Lef1, a transcriptional activator and a target gene of the Wnt/β-catenin pathway. These Lef1-positive;GFP-negative cells expressed the telencephalic marker Foxg1 but not Ascl1, which is normally expressed by ventral telencephalic cells. These results suggest that the EmT has the capacity to activate Wnt/β-catenin signalling in the ventral telencephalon and to suppress ventral telencephalic gene expression. Altogether, our data support a role of the EmT as a signalling centre in the developing mouse forebrain.

**Electronic supplementary material:**

The online version of this article (doi:10.1007/s00429-015-1127-3) contains supplementary material, which is available to authorized users.

## Introduction

The specification and organization of the central nervous system (CNS) start at the gastrula stage of embryonic development. Following the induction of its primordium, the neural plate, by the Spemann–Mangold organizer, the CNS is coarsely patterned along its anteroposterior and dorsoventral axes (reviewed by Kiecker and Lumsden 2012). Neural induction and neural patterning in vertebrates are mediated through signalling molecules (secreted proteins) that emanate from signalling centres (Grove et al. 1998; Houart et al. 1998; Martinez 2001; Martinez and Alvarado-Mallart 1989; Shimamura and Rubenstein 1997).

Local signalling centres are transient structures that usually lie at the boundaries of the tissues they pattern, and secrete molecules that diffuse through adjacent tissue to establish gradients that confer positional information on the cells (Grove and Fukuchi-Shimogori 2003; Kiecker and Lumsden 2005; Wolpert 1996, 2011). Also, in a manner similar to the early organizer, these local signalling centres possess the ability to induce ectopic cell fates in host tissues when heterotopically transplanted (Liu and Joyner 2001; Martinez et al. 1991; Raible and Brand 2004; Shimamura and Rubenstein 1997). The recent combination of genetic and molecular methods with morphological approaches led to the identification of three such local signalling centres in the developing forebrain: the anterior neural ridge (ANR), the cortical hem (hem) and the zona limitans intrathalamica (ZLI). The ANR is located in the most rostral part of the neural plate and is a source of Fgf molecules. Ablation of the neural ridge leads to loss of anterior forebrain fates in both mouse and zebrafish (Houart et al. 1998, 2002; Shimamura and Rubenstein 1997). The hem is located in the dorsomedial telencephalon and is rich in expression of Wnt and Bmp molecules (Grove et al. 1998; Lee et al. 2000). It is essential for the specification and patterning of the hippocampal primordium. *Gli3*^−*/*−^ mutant mice (*extratoes*) with no hem tissue do not develop a hippocampus (Theil et al. 1999; Tole et al. 2000), while *Lhx2*-null mouse chimeras develop ectopic hems that induce the formation of multiple hippocampal fields (Mangale et al., 2008; Subramanian and Tole 2009). The ZLI is found in the diencephalic primordium separating the prethalamus from the thalamus. It secretes Shh and is crucial for diencephalic specification and growth (Kiecker and Lumsden 2004; Martinez-Ferre and Martinez 2012; Vieira et al. 2005).

The eminentia thalami (EmT) is a forebrain structure that forms, along with the prethalamus, in prosomere 3 (p3) of the prosencephalon (Puelles and Rubenstein 2003). It is flanked medially by the prethalamus and laterally by the choroid plexus of the lateral ventricle. Its rostral part forms the posterior limit of the interventricular foramen of Monroe, and the caudal part becomes attached to the ventral telencephalon (Abbott and Jacobowitz 1999; Trujillo et al. 2005). The role of the EmT in mammals has not been fully determined. It is postulated that the EmT may be a source of a subtype of Cajal-Retzius cells in the developing neocortex (Meyer 2010; Tissir et al. 2009). Based on its location at the interface between the diencephalon and telencephalon and its transient nature, Abbott and Jacobowitz (1999) suggested that it may act as an organizing centre in forebrain patterning. A recent report proposes that the EmT forms part of the forebrain hem system and may play a role in forebrain patterning and development (Roy et al. 2014).

In this study, we aimed to shed light into the putative role of the EmT as a forebrain signalling centre. Using an extensive battery of signalling molecules, we first examined the expression of members of the Wnt, Fgf and Bmp families in the EmT from E11.5 to E17.5, to determine their temporal and spatial patterns of expression. Members of these developmental control gene families have been implicated in patterning other parts of the CNS (Grove et al. 1998; Shimamura and Rubenstein 1997; Liu and Joyner 2001; Martinez 2001; Martinez et al. 1991; Houart et al. 2002). The EmT was found to express several members of these families, with Wnts being the most broadly and intensely expressed signalling molecules. Next, we examined whether the EmT had the capacity to induce ectopically cell fate changes in the ventral telencephalon when heterotopically transplanted. We observed that the EmT, but not neocortical tissue, was able to induce in ventral telencephalic cells ectopic expression of Lef1, a target gene and transcriptional activator of the Wnt/β-catenin pathway. These Lef1-positive cells expressed the telencephalic marker Foxg1, but were negative for the ventral telencephalic marker Ascl1. Our data explore the molecular and cellular properties of the EmT and support the notion that this structure may act as a signalling centre in the mouse developing forebrain.

## Materials and methods

### Animals

Animal care was according to institutional guidelines. Mice were mated, and the morning of the vaginal plug was designated as embryonic day (E) 0.5. Time-mated pregnant mice were culled by cervical dislocation or overdose of Isoflurane (Merial, UK). Embryos used for gene expression analyses were obtained from wild type female mice of CD1 background. The donor eminentia thalami tissue was from tau-GFP transgenic mouse embryos (Pratt et al. 2000). Embryos were dissected out of the uteri in ice-cold phosphate-buffered saline (PBS). For culture experiments, embryos were dissected in ice-cold 1 × Kreb’s buffer.

### Organotypic slice culture

Organotypic slice cultures of the E13.5 embryonic mouse forebrain were done as previously described (Magnani et al. 2010). Brains were removed and placed into ice-cold 1 × Kreb’s buffer with 10 mM HEPES buffer (Invitrogen), Gentamicin (Sigma) and Penicillin–Streptomycin (Invitrogen). Brain tissue was embedded in molten 4 % Low Melting Point agarose (Seakem) in PBS at 43°C with stirring and was solidified on ice. Tissue was sectioned coronally on a vibratome at a thickness of 300 μm. Slices were collected into chilled 1 × Kreb’s buffer with HEPES and antibiotics. Brain slices were cultured in organ tissue dishes containing 1 ml of medium (Neurobasal, Gibco) supplemented with B-27, glutamine, glucose, penicillin, and streptomycin. For transplantations, brain slices were cultured in Millicell-CM 30 mm low height membrane inserts (Millipore). Eminentia thalami were dissected out from one or both sides of the brain of donor slices, and transplanted to the ventral telencephalon of host slices. Slices were re-incubated for between 24 and 48 h, after which they were fixed in 4 % paraformaldehyde (PFA) in 0.1 M phosphate buffer (PB) overnight at 4°C and processed for immunohistochemistry or in situ hybridization. For activation of Wnt/β-Catenin signalling in the ventral telencephalon, slices were cultured on polycarbonate culture membranes (8 μm pore size; Corning Costar) in the presence of either 1 % dimethyl sulfoxide (DMSO) or of 5, 25 or 50 μM CHIR99021 (CHIR) (Cambridge BioScience). Slices were cultured for 24 h, fixed as described above, and processed for immunohistochemistry or in situ hybridization as described below.

### Histology, immunohistochemistry, in situ hybridization

Embryos for gene expression analysis were collected from E11.5 to E17.5. At least two embryos were analysed for each age. Embryos were decapitated and the heads were immersion fixed in 4 % PFA in 0.1 M PB overnight at 4°C. Cultured slices were fixed as described above.

Fixed tissues were processed for immunohistochemistry or in situ hybridization following previously described protocols (Fotaki et al. 2006, 2013).

For DAB and fluorescence immunodetection, antigen retrieval was achieved by microwaving sections in 10 mM sodium citrate buffer. Following DAB reaction, some slides were counterstained with 0.5 % cresyl violet acetate (Sigma). For immunofluorescence, species-specific secondary antibodies conjugated to Alexa fluor-488, 568 or 647 dyes (1:200, Invitrogen) were used to detect primary antibodies. When signal amplification was required, sections were incubated with biotinylated secondary antibodies. Antibody reactions were then revealed using streptavidin–biotin complex conjugated to either 488 or 568 dyes (1:200, Invitrogen). Sections were counterstained with DAPI (1:10000, Sigma). Antibodies used in this study were mouse monoclonals for Ascl1 (1:200, BD Biosciences), Lhx1 and Lhx5 (Lim1+2; 1:100, DSHB) and Pax6 (1:200, DSHB); rabbit polyclonals for Calretinin (1:1000, Swant), Lef1 (1:1000, Cell Signalling) and Tbr2 (1:1000, kindly provided by Prof. R. Hevner); a goat polyclonal for GFP (1:500, Abcam) and a chick polyclonal for GFP (1:200, AVES Labs).

For in situ hybridization, 1 μg of each linearized purified DNA template was transcribed using T3, T7 or SP6 RNA polymerase. RNA antisense probes were labelled using the digoxygenin RNA labelling mix (Roche) according to the manufacturer’s instructions. The following riboprobes were used in this study: *Bmp4*, *Bmp6*, *Fgf8*, *Fgf15*, *Mkp3*, *Spry1*, *Wnt3a*, *Wnt7a*, *Wnt7b*, *Wnt8b*, *Wnt9a*, *Axin2*, *Lef1*, *Sfrp2*, *Ngn2* and *Foxg1*.

### Quantitation of Lef1-positive and Ascl11-positive cells

To determine the effect of the different concentrations of CHIR on Lef1 induction and upregulation as well as Ascl1 downregulation, Lef1-positive, Ascl1-positive and Lef1;Ascl1-positive cells were counted in five representative sections of every slide using the Image J 1.46r software (NIH) and the mean determined (from fifteen sections counted from three slices) for each of the concentrations used. Means of the various concentrations were then compared by a one-way Anova using the Graphpad Instat3. *P* values less than 0.05 were considered significant.

## Results

### Analysis of expression of molecular markers of the EmT

**Fig. 1:**
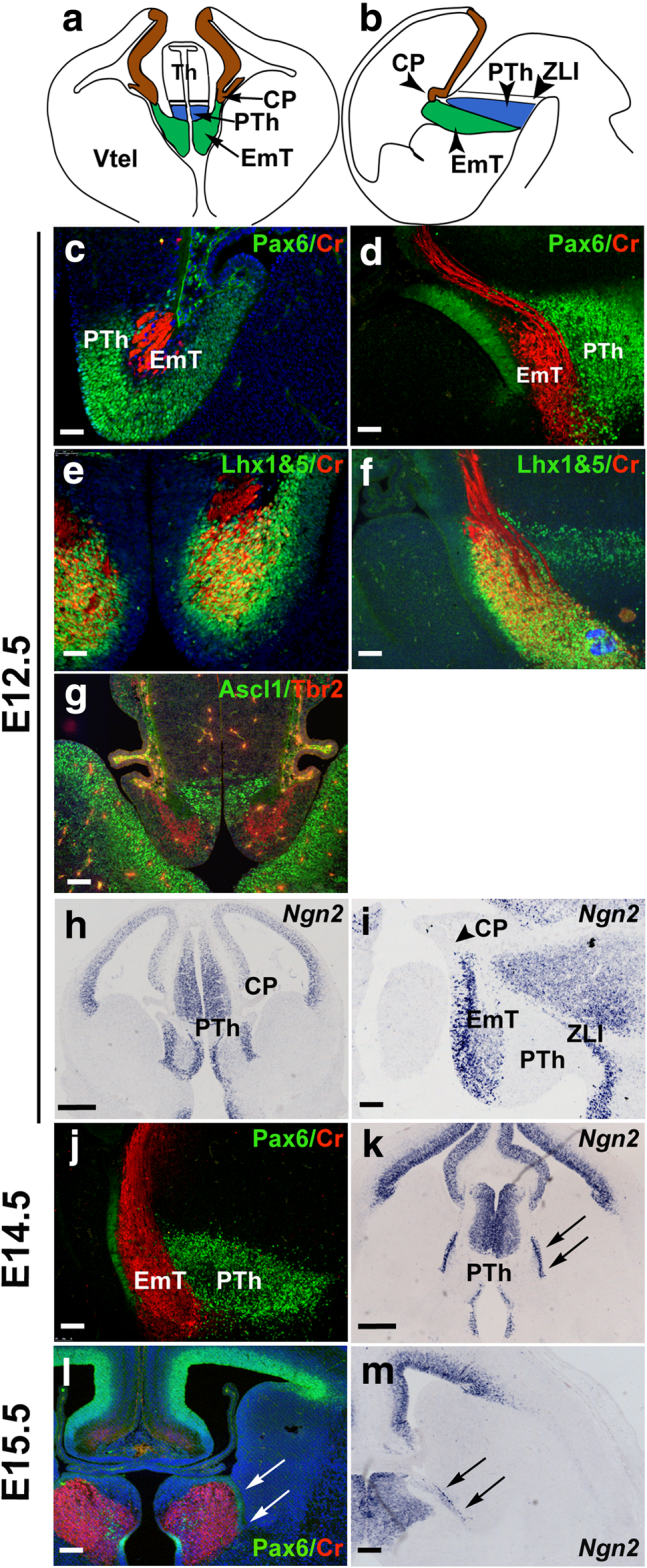
Expression of molecular markers of the EmT. **a**, **b** Schematic representations of a coronal (**a**) and a sagittal section (**b**) of an E12.5 forebrain. The prethalamus (*blue*) is located dorsal (**a**) and medial (**b**) to the EmT (*green*). Brown staining outlines the dorsomedial telencephalon. In **b** and all following sagittal sections (**d**, **f**, **i**, **j**), rostral is to the *left* and dorsal is to the *top*. **c**, **d** Pax6 (*green*) and Calretinin (CR) (*red*) double immunofluorescence showing expression of these proteins in the EmT at E12.5. In both coronal (**c**) and sagittal (**d**) sections, Pax6 is expressed in the proliferative layer while CR is expressed by the postmitotic cells and fibres of the EmT. Pax6 is also expressed in the prethalamus. **e**, **f** Lhx1&5 (*green*) and CR (*red*) immunofluorescence reveals strong Lhx1&5 expression in both the proliferative and differentiating layers of the EmT at E12.5 in both coronal (**e**) and sagittal sections (**f**). **g** Ascl1 (*green*) and Tbr2 (*red*) immunofluorescence on coronal E12.5 sections reveals Tbr2 expression within the EmT. Ascl1 is found in the adjacent prethalamus. **h**, **i** *In situ* hybridization for *Ngn2* at E12.5 in both coronal (**h**) and sagittal (**i**) sections. At this stage, *Ngn2* mRNA expression distinguishes the EmT from both the prethalamus medially and choroid plexus (*arrowhead*) laterally. **j**, **l** Expression of Pax6 (*green*) and CR (*red*) in the EmT in a sagittal section at E14.5 (**j**) and a coronal section at E15.5 (**l**). Pax6 and CR still mark the proliferative and postmitotic layers, respectively. **k**, **m** *Ngn2* expression is still seen in the ventricular zone laterally (*arrows*) and medially, marking its boundary with the choroid plexus and prethalamus, respectively, both in a coronal section at E14.5 (**k**) and E15.5 (**m**). *Blue* colour in (**l**) is DAPI used as counterstain. Abbreviations: *CP* choroid plexus, *PTh* prethalamus, *Th* thalamus, *Vtel* ventral telencephalon, *ZLI* zona limitans intrathalamica. *Scale bars* h, k = 250 μm; g, i, l and m = 100 μm; c–f, j = 50 μm

The EmT is a transient structure of the developing mammalian forebrain, which, in a coronal plane, is flanked dorsomedially by the prethalamus and laterally by the choroid plexus (Fig. 1a and Online Resource 3), while in a sagittal plane the EmT lies caudal to the choroid plexus and rostral to the prethalamus (Fig. 1b). To demarcate the EmT and gain a better insight into how it changes during development, we performed immunofluorescence and in situ hybridization using markers that are expressed in this structure at different developmental stages. At E12.5, double immunofluorescence for Pax6 and Calretinin revealed strong Pax6 staining in the ventricular zone (VZ) of the EmT and the prethalamus and Calretinin staining in the EmT cell fibres (Fig. 1c, d) (Abbott and Jacobowitz 1999; Fotaki et al. 2006; Retaux et al. 1999). Double immunofluorescence with an antibody that recognises both Lhx1 and Lhx5 and Calretinin revealed strong expression of Lhx1&5 in the layer of differentiated neurons of the EmT that was also positive for Calretinin as well as the VZ of the EmT (Fig. 1e, f). Double immunofluorescence for the transcription factors Tbr2 and Ascl1 revealed Tbr2 expression in the layer of differentiated neurons of the EmT, while Ascl1 expression was restricted to the prethalamus (Fig. 1g). *Ngn2* mRNA staining was found at the VZ of the EmT but was not detected in the adjacent prethalamus (Flames et al. 2007), thus allowing us to distinguish the medial boundary of the EmT (Fig. 1h, i).

At E14.5, the VZ of the EmT marked by strong Pax6 protein and *Ngn2* mRNA was significantly thinner than that observed at younger stages (Fig. 1j, k compare 1d to 1 j and 1 h to 1 k). At E15.5 and E16.5, Pax6 and *Ngn2* expression in this zone was each detected as a stripe of staining ventral to the choroid plexus at all rostrocaudal levels (arrows in Fig. 1l, m and not shown).

### Expression of *Wnt*, *Fgf* and *Bmp* genes in the EmT

The hypothesis that the EmT acts as a signalling centre predicts that it expresses one or more signalling molecules. To examine this, we analysed the expression of members of the Wnt, Fgf and Bmp families of signalling molecules.

We first examined the expression of members of the Wnt family of signalling molecules. Out of the nineteen members, we examined the expressions of *Wnt3a*, *Wnt7a*, *Wnt7b*, *Wnt8b* and *Wnt9a* which were more likely to be expressed in the EmT based on results from database searches (Genepaint: http://genepaint.org/; The Jackson Laboratory: http://www.jax.org/; Allen Brain atlas: http://www.brain-map.org/; EMAGE: http://emouseatlas.org/emage/). In our study, we also included *Axin2* and *Lef1* which are downstream target genes of the pathway, and *Sfrp2*, a negative regulator of the pathway (Hsu et al. 1998; Jho et al. 2002; Porfiri et al. 1997). Expression was studied along the rostrocaudal axis from E11.5 until gene expression was not evident. Unless otherwise stated, gene expression for each specific gene was found along the entire axis.

**Fig. 2:**
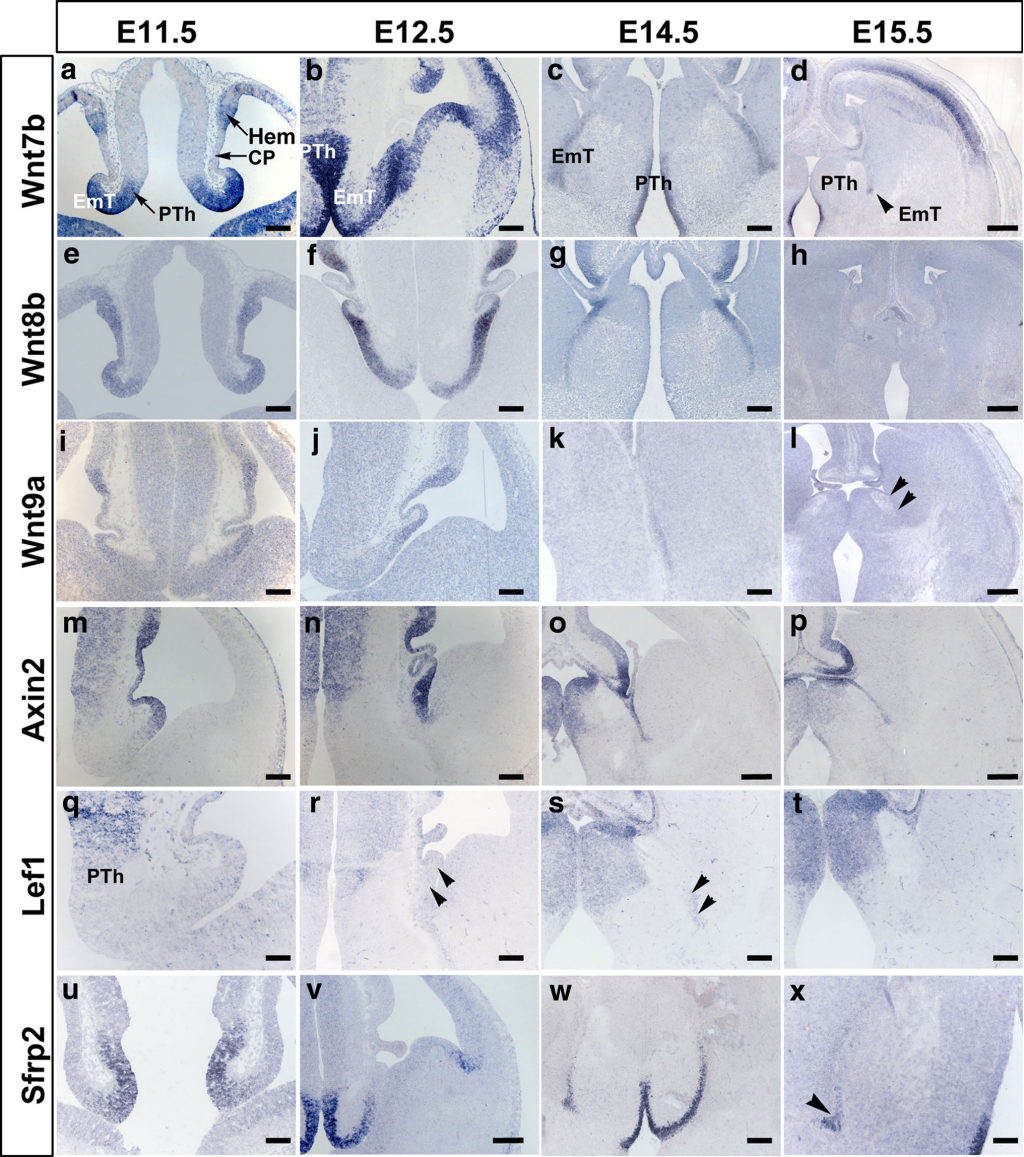
Expression of Wnt and Wnt-related genes in the EmT from E11.5. **a**–**d** *Wnt7b* expression. Strong *Wnt7b* mRNA is detected in the EmT at E11.5 (**a**) and 12.5 (**b**) and is downregulated at E14.5 (**c**) and E15.5 (*arrowhead* in **d**). **e**–**h** *Wnt8b* expression. Strong *Wnt8b* mRNA is detected from 11.5 to 14.5 (**e**–**g**), after which it is no longer detectable in the EmT (**h**). **i–l** *Wnt9a* expression. Weak *Wnt9a* mRNA is detected in the caudo-lateral EmT from E11.5 to E14.5 (**i**–**k**), after which it is no longer detected (*arrowheads* in **l** mark the lateral limit of the EmT at E15.5). **m–p** *Axin2* expression. *Axin2* mRNA expression is seen in the lateral aspect of the EmT from E11.5 to E15.5. **q–t** *Lef1* expression. At E11.5 (**q**), *Lef1* mRNA is not detected in the EmT, but it is found in the prethalamus medial to it. From E12.5 to E14.5 (**r**, **s**), very weak Lef1 mRNA is detected in the lateral aspect of the EmT (*arrowheads*). This weak expression is not detected from E15.5 onwards (**t**). **u–x** *Sfrp2* expression. Strong *Sfrp2* mRNA is detected in the EmT from E11.5 to E14.5 (**u**–**w**), and becomes weak at E15.5 (*arrowhead* in **x**). Abbreviations: *CP* choroid plexus, *PTh* prethalamus, *Th* thalamus, *Vtel* ventral telencephalon. *Scale bars* d, h, l, p = 200 μm; a–c, e–g, i–k, m, n, r–t, v, w = 100 μm; u, x = 50 μm; q = 25 μm

Results of in situ hybridization on E11.5 forebrain tissue showed that at this stage the EmT expresses most of the Wnt and Wnt-related genes analysed. *Wnt7b* mRNA expression in the EmT was strong and involved the entire thickness of the neuroepithelium. It extended from its medial extent, where it was continuous with a similar strong expression in the prethalamus, towards its lateral aspects (Fig. 2a). Expression was detected in the cortical hem of the dorsomedial telencephalon, but not in the adjacent choroid plexus of the lateral ventricle (Fig. 2a). *Wnt8b* mRNA was detected as a strong homogenous staining involving the neuroepithelium of the EmT, the choroid plexus of the lateral ventricle, the cortical hem and the rest of the medial pallium (Fig. 2e). Expression in the EmT appeared strong throughout, except at its most medial extent bordering the prethalamus (Fig. 2e). Weak *Wnt9a* mRNA was detected in the lateral part of the caudal EmT (Fig. 2i). *Wnt3a* and *Wnt7a* were not detected at this stage (not shown). *Axin2* mRNA expression in the EmT was strong but was found only in the most lateral aspect of the EmT (Fig. 2m). This strong expression was continuous with a similar expression in the developing choroid plexus and cortical hem in the dorsomedial telencephalon. *Sfrp2* expression formed a high-medial to low-lateral gradient, but did not involve the most lateral aspects bordering the choroid plexus (Fig. 2u). *Lef1* mRNA was not detected in the EmT at this stage, but some mRNA was detected in the prethalamus medial to it (Fig. 2q).

At E12.5, the genes detected at E11.5 were still expressed in the EmT. The pattern and level of expression of most of these genes at this stage were similar to those observed at E11.5 (Fig. 2b, f, j, n, v). However, *Wnt8b* mRNA expression in the EmT was no longer homogeneous but formed a gradient with the highest levels detected in the lateral EmT, and the lowest level in the medial part bordering the prethalamus (Fig. 2f). Also, there was what appeared to be a very weak *Lef1* mRNA expression in the caudo-lateral part of the EmT at this stage (Fig. 2r). In addition, weak *Wnt3a* expression was detected in the most lateral part of the EmT adjacent to the choroid plexus in the middle and caudal sections (not shown). *Wnt7a* was also detected at this stage in middle and caudal sections but, unlike *Wnt3a*, it was detected in the medial half of the EmT (not shown).

At E14.5, most expression patterns were similar to those observed at E12.5 (Fig. 2c, g, k, o, s, w). However, for *Wnt7b*, Wnt*8b* and Wnt*9a*, the strength of staining appeared lower than that observed at younger stages (Fig. 2c, g, k). The VZ marked by *Wnt7b*, *Wnt8b*, *Axin2* and *Sfrp2* also appeared thinner (Fig. 2c, g, o, w). *Wnt3a* and *Wnt7a* were not detected in the EmT at E14.5 (not shown).

Expression of *Wnt7b* mRNA was very weak in the EmT at E15.5 (Fig. 2d) and E16.5 (not shown) and was not identifiable at E17.5. *Wnt8b*, *Wnt9a* and *Lef1* mRNA were not detected in the EmT from E15.5 onwards (Fig. 2h, l, t). Expression of *Axin2* and *Sfrp2* mRNAs in the EmT at E15.5 appeared weaker than that observed at E14.5 (Fig. 2p, x) and was not detected beyond E15.5 (not shown).

All the above *Wnt* and *Wnt*-related genes were expressed in the VZ. *Wnt7b* showed an additional weak expression in the mantle layer at E12.5 (Fig. 2b).

**Fig. 3:**
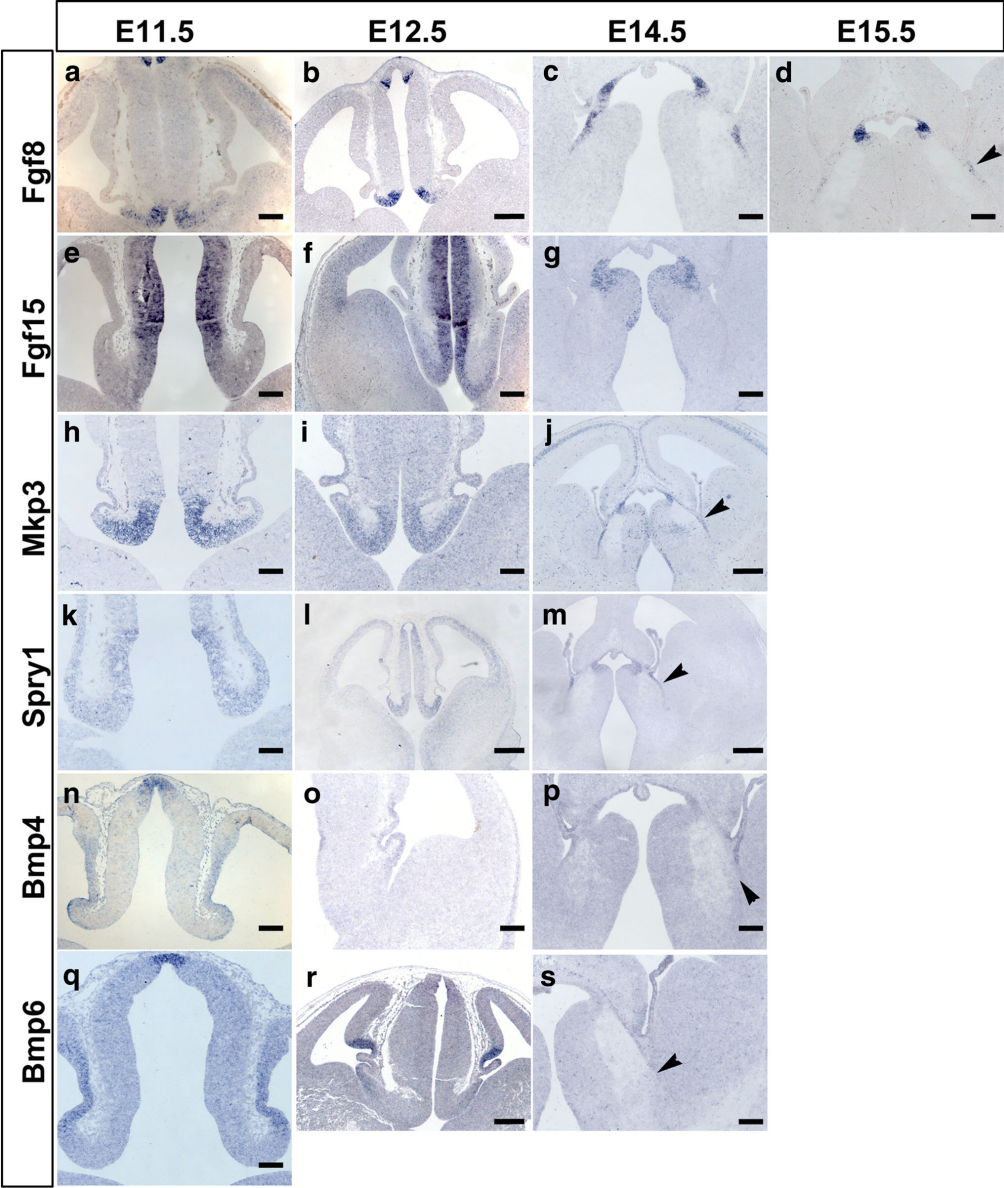
Expression of Fgf and Bmp-related genes in the EmT from E11.5 to E15.5 **a–d** *Fgf8* expression. Strong *Fgf8* mRNA expression detected in the EmT at E11.5 (**a**) and 12.5 (**b**), is downregulated at E14.5 (**c**) and E15.5 (**d**). **e–g** *Fgf15* expression. *Fgf15* mRNA is detected in the middle and caudal EmT at E11.5 (**e**) and E12.5 (**f**) but is no longer detectable in the EmT at E14.5 and beyond (**g**). **h**–**j** Strong *Mkp*3 mRNA is expressed in the entire rostrocaudal extent of the EmT till E14.5. Sections shown are from the *middle portion* of the EmT. **k**–**m** *Spry1* mRNA is expressed in the EmT from E11.5 to E14.5. **n**–**p** *Bmp4* mRNA expression is detected in the most lateral tip of the EmT till E14.5. **q**–**s** *Bmp6* mRNA is expressed in the lateral EmT at E11.5 and E12.5 but not at E14.5 (*arrowhead* in **s**). *Scale bars* b, j, l, m, r = 200 μm; a, c–i, k, n–p, q, s = 100 μm

We then examined expression of members of the Fgf and Bmp signalling pathways in the EmT (Fig. 3). Based on the results using the same databases as for the study of *Wnts*, we restricted our analysis to expression of two members of each family: *Fgf8* and *Fgf15* for the Fgfs (Fig. 3a–g) and *Bmp4* and *Bmp6* for the Bmps (Fig. 3n–s). We also included in our study expression of *Mkp3* and *Spry1*, which are downstream targets of Fgf signalling (Mason 2007; Suzuki-Hirano et al. 2010) (Fig. 3h–m).

At E11.5, the intensity of *Fgf8* mRNA staining was high in the rostral EmT (Fig. 3a), but low in the middle and absent in the caudal EmT (not shown). Weak *Fgf15* mRNA expression was detected in the middle and caudal parts of the medial EmT (Fig. 3e), but not in the rostral part. *Mkp3* mRNA expression in the EmT was strong at all rostrocaudal levels and appeared to form a high-medial to low-lateral gradient (Fig. 3h). *Spry1* mRNA was also found in the entire neuroepithelium at all rostrocaudal levels of the EmT, although expression was weak (Fig. 3k). The above molecules were not detected in the neighbouring choroid plexus and cortical hem. *Bmp4* and *Bmp6* were expressed in the EmT, as well as the cortical hem, choroid plexus and epithalamus. Expression in the EmT was weaker than that detected in the epithalamus, and was confined to its most lateral aspect bordering the choroid plexus (Fig. 3n, q).

At E12.5, the above described *Fgf* and *Bmp* genes were still expressed in the EmT (Fig. 3b, f, i, l, o, r). Moreover, the patterns of expression of these genes were similar to those observed at E11.5. At E14.5, *Fgf8*, *Mkp3*, *Spry1* and *Bmp4* were still expressed in the EmT (Fig. 3c, j, m, p). However, the intensity of staining seen at this stage appeared weaker and the VZ marked by these genes was also thinner than seen at younger ages. *Fgf15* and *Bmp6* were not detected in the EmT at this stage (Fig. 3g, s). At E15.5, only *Fgf8* was still detected in the EmT (Fig. 3d).

To summarize, all the above members of the Wnt/β-catenin signalling pathway were found in the VZ of the EmT, where staining decreased as development progressed and ceased for most of them by E15.5. Similar results were obtained for the molecules of the Fgf and Bmp signalling pathways, except that most of them were not detected beyond E14.5. The above results are summarized in Table 1.

**Table 1:** Summary of gene expression in the EmT along the rostrocaudal axis

		*Bmp4*	*Bmp6*	*Fgf8*	*Fgf15*	*Mkp3*	*Spry1*	*Wnt7b*	*Wnt8b*	*Wnt9a*	*Axin2*	*Lef1*	*Sfrp2*
*E11.5*	*R*	+	+	++	n.d	++	+	+++	+++	n.d	++	n.d	++
	*M*	+	+	+	+	++	+	+++	+++	n.d	++	n.d	++
	*C*	+	+	n.d	+	++	+	+++	+++	+	++	n.d	++
*E12.5*	*R*	+	+	++	n.d	++	++	+++	+++	n.d	++	n.d	+++
	*M*	+	+	+	+	++	++	+++	+++	n.d	++	n.d	+++
	*C*	+	+	n.d	+	++	++	+++	+++	+	++	+	+++
*E14.5*	*R*	+	n.d	+	n.d	+	+	++	++	n.d	++	n.d	++
	*M*	+	n.d	+	n.d	+	+	++	++	n.d	++	n.d	++
	*C*	+	n.d	n.d	n.d	+	+	++	++	+	++	+	++
*E15.5*	*R*	n.d	n.d	+	n.d	n.d	n.d	+	n.d	n.d	+	n.d	+
	*M*	n.d	n.d	+	n.d	n.d	n.d	+	n.d	n.d	+	n.d	+
	*C*	n.d	n.d	n.d	n.d	n.d	n.d	+	n.d	n.d	+	n.d	+

Legend: *R* rostral, *M* middle, *C* caudal, *n.d*. not detected, *+* weak, *++* strong, *+++* very strong

### Effects of small molecule activation of Wnt/β-catenin signalling in the ventral telencephalon

Analyses of gene expression in the EmT showed some distinct and some overlapping expression domains of *Wnt*, *Fgf* and *Bmp* genes. The *Wnt* genes showed stronger expression during EmT development compared to those of the *Fgf* and *Bmp* families. For this reason, we chose to follow up on the role of Wnt/β-catenin signalling pathway in EmT function.

To examine whether the Wnt molecules expressed in the EmT have a signalling capacity, we transplanted EmT tissue into the ventral telencephalon to investigate if Wnt signals from the EmT were able to induce any cell fate changes in this region (see following section). We chose the ventral telencephalon as it normally does not express significant levels of Wnt/β-catenin target genes (Backman et al. 2005; Fotaki et al. 2011; Maretto et al. 2003).

Our first step was to test whether the ventral telencephalon was competent to respond to Wnt/β-catenin signalling. For this, we activated the pathway in telencephalic slices by treating them in culture with three different concentrations of CHIR 99021 (CHIR) (5, 25 and 50 µM) in 1 % Dimethyl sulfoxide (DMSO) to dose-dependently inhibit GSK3β activity. CHIR is a substituted dihydropyromidine whose ability to inhibit GSK3β facilitates the cytoplasmic accumulation and subsequent translocation to the nucleus of β-catenin, which is required for the transcription of downstream targets of the Wnt/β-catenin pathway (Ring et al. 2003). Our controls were slices that were treated in culture with 1 % DMSO.

**Fig. 4:**
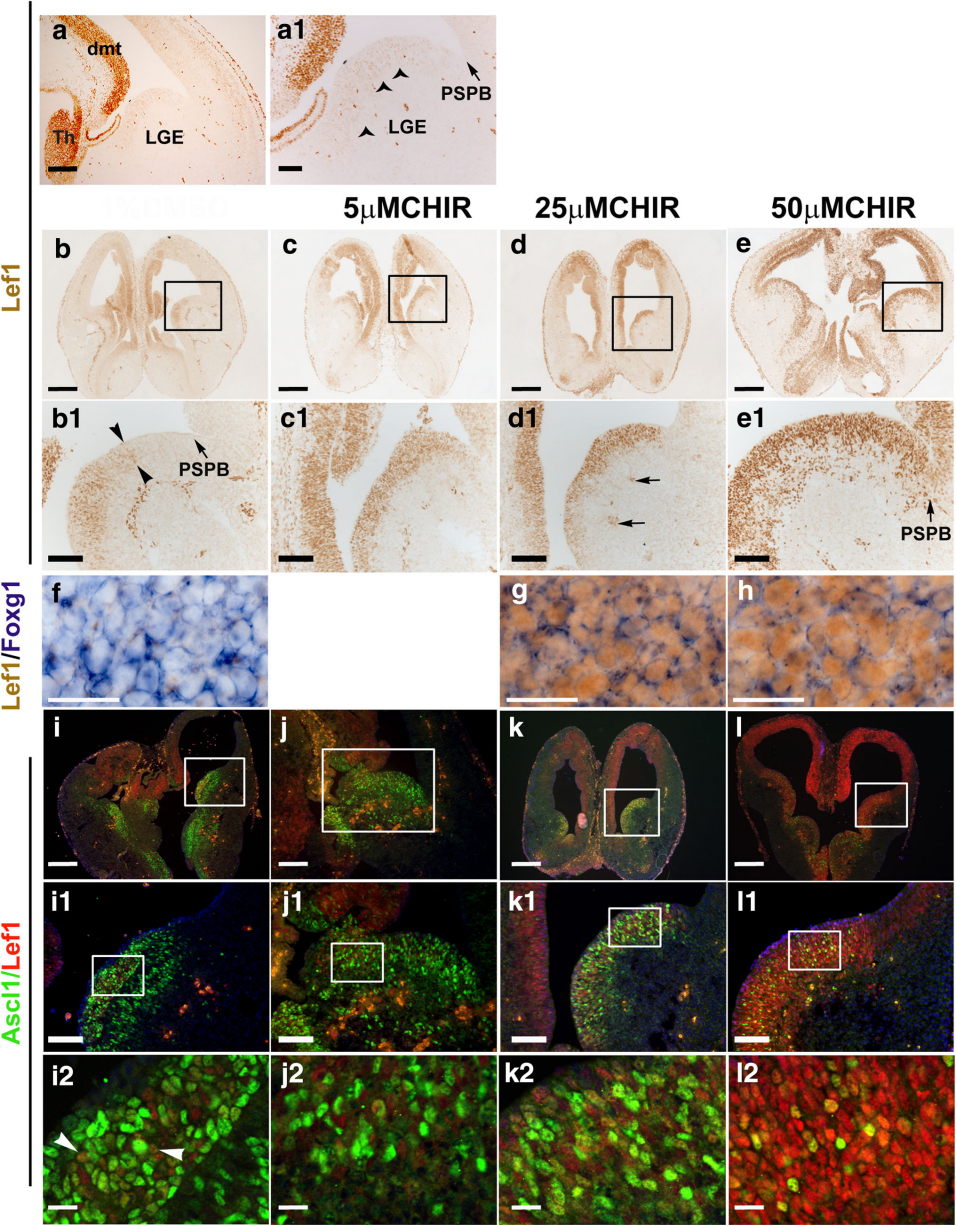
Effect of Wnt/β-catenin signalling upregulation on gene expression in the ventral telencephalon. **a**, **a** ^**1**^ Lef1 expression in the ventral telencephalon of CD1 mice at E 13.5. Lef1 is strongly expressed in the dmt and Th. *Arrowheads* in a^1^ point to faintly expressing Lef1-positive cells detected in the VZ of the ventral telencephalic LGE and at a distance from the PSPB (indicated by an *arrow*). a^1^ is a magnification of a. **b**–**e** Lef1 expression in the ventral telencephalon of treated slices. **b**, **b** ^**1**^ Lef1 expression in DMSO-treated slices. Lef1 is detected in parts of the VZ and superficial mantle layer. Staining is not seen at the PSPB but at some distance away from it, as shown with *arrowheads* in b^1^. **c**, **c** ^**1**^ Lef1 expression in slices treated with 5 μM CHIR and (**d**, **d** ^**1**^) 25 μM CHIR. A fairly strong Lef1 expression is seen in the ventral telencephalon. Lef1 expression marks almost the entire VZ and superficial mantle layer beginning from the subpallial side of the PSPB. The *arrows* in d^1^ point to blood vessels found in the mantle. **e**–**e** ^**1**^ Lef1 expression in 50 μm CHIR-treated slices. Very strong Lef1 expression is detected in the ventral telencephalon. Several Lef1-positive cells are seen in the VZ and mantle layer of the ventral telencephalon. In the VZ, Lef1 is also expressed in the PSPB (*arrowhead* in e^1^). b^1^, c^1^, d^1^ and e^1^ are magnifications of the regions indicated in b, c, d and e, respectively. **f**–**h** Lef1 and *Foxg1* expression in treated slices. In both control (**f**) and CHIR-treated groups (**g**, **h**) *Foxg1*-positive cells (*blue*-in situ hybridization staining) in the ventral telencephalon also express Lef1 (*brown*-DAB staining). **i**–**l** Lef1 and Ascl1 expression in the ventral telencephalon of treated slices. **i**, **i** ^**1**^ and **i** ^**2**^ In DMSO-treated slices, Ascl1 is detected in the VZ and mantle layer of the ventral telencephalon. A few Lef1-positive cells are detected within these zones (**i**, **i** ^1^). Most of the Lef1-positive cells are Ascl1-negative and only a few are double-labelled (*arrowheads* in i^2^). **j**, **j** ^**1**^ and **j** ^**2**^ Lef1 and Ascl1 expression in 5 μM CHIR-treated slides. Several Ascl1-expressing cells are detected in the VZ and mantle. The intensity of staining seen in many of the cells is similar to that seen in i. Many of the cells are single-labelled (**j** ^2^). **k**, **k** ^**1**^ and **k** ^**2**^ Lef1 and Ascl1 expression in 25 μM CHIR-treated slides. Fewer Ascl1-expressing cells than those detected in i and j are seen in the VZ and mantle layer. **l**, **l** ^**1**^ and **l** ^**2**^ Lef1 and Ascl1 expression in 50 μM CHIR-treated slides. Very few Ascl1-positive cells are seen in the VZ and mantle layer of the ventral telencephalon. On the other hand, many Lef1-expressing cells are detected in these zones, many of which are Ascl1-negative. Abbreviations: *dmt* dorsomedial telencephalon, *LGE* lateral ganglionic eminence, *PSPB* Pallial–Subpallial Boundary, *Th* Thalamus, *VZ* Ventricular zone. *Scale bars* b–e, k–n = 250 μm; a, b^1^–e^1^, i^1^–l^1^ = 50 μm, a^1^, f–h, i^2^–l^2^ = 20 μm

Lef1 is a transcriptional activator in the Wnt/β-catenin signalling pathway (Hsu et al. 1998; Porfiri et al. 1997) and is expressed in the thalamus and the dorsal and dorsomedial telencephalon (Fig. 4a, a^1^) (Fotaki et al. 2011; Galceran et al. 2000). DAB immunohistochemistry with an antibody for Lef1 revealed a small number of Lef1-positive cells in the VZ of the lateral ganglionic eminence (LGE), located at a distance from the pallial–subpallial boundary (PSPB) (Fig. 4a, a^1^). These cells express very low levels of Lef1 compared to the Lef1 heavily stained cells in the dorsomedial telencephalon (Fig. 4a^1^) and were not detectable using Lef1-immunofluorescence (not shown). To determine the level of activation of Wnt/β-catenin signalling in the ventral telencephalon, we analysed in both control and experimental slices Lef1 expression in this region.

In DMSO-treated slices, Lef1 expression was detected in the VZ of the LGE (Fig. 4b, b^1^) at a similar position to that described above (Fig. 4a, a^1^). However, expression was stronger compared to non-treated sections (compare Fig. 4b^1^ to 4a^1^), but it was still lower than Lef1 expression in the dorsomedial telencephalon from the same section (Fig. 4b, b^1^). A mean of 245.13 (±SD = 38.02) Lef1-positive cells per section was counted in the ventral telencephalon of these control slices (Table 2).

**Table 2:** Lef1-positive cells in the ventral telencephalon of treated slices

Lef1-positive cells per section				
	Mean	STDEV	% increase	*P* value
DMSO	245.13	38.02	N/A	N/A
5 μM CHIR	340.80	45.56	39.03	<0.001
25 μM CHIR	391.33	37.87	59.64	<0.001
50 μM CHIR	715.80	102.18	192.00	<0.001

Shown are the mean number of cells per section with the standard deviation (STDEV), the  % increase in number of Lef1-positive cells compared to the control (DMSO) group and the *P* values of each group compared to the control

In both 5 μM and 25 μM CHIR-treated slices, strong Lef1 expression was detected in the VZ and the superficial mantle layer of the ventral telencephalon (Fig. 4c, d). The intensity of staining seen in the ventral telencephalon at these two concentrations was similar to each other and appeared higher than that seen in DMSO-treated slices (compare Figs. 4c^1^ and d^1^ to 4b^1^). Cell counts revealed a statistically significant increase of 39 % (*P* < 0.001) and 60 % (*P* < 0.001) in Lef1-positive cells in slices treated with 5 and 25 μm CHIR, respectively, compared to the control group (Table 2). In 50 μM CHIR-treated slices, very strong Lef1 expression was detected in the VZ as well as in the mantle layer of the ventral telencephalon (Fig. 4e, e^1^). Moreover, Lef1 expression in the VZ involved its entire mediolateral extent, and included the PSPB (Fig. 4e^1^). Numbers of Lef1-positive cells in the ventral telencephalon were ~192 % greater than those found in the control (*P* < 0.001) (Table 2). These results confirm that the ventral telencephalon is competent to respond to Wnt/β-catenin signalling.

We then examined the expression of Foxg1 and Ascl1 to study how the increase in Lef1 staining in the ventral telencephalon may affect distribution of these proteins in this region. In wild types after E12.5, Foxg1 is detected at high levels in both the dorsal and ventral telencephalon (Fotaki et al. 2006; Hanashima et al. 2002; Tao and Lai 1992; Xuan et al. 1995), while Ascl1 is expressed at high levels in the ventral telencephalon (Guillemot and Joyner 1993; Lo et al. 1991; Porteus et al. 1994).

In both DMSO and CHIR-treated slices, *Foxg1* was expressed in the entire ventral telencephalon forming a gradient, with expression in the VZ being higher than expression in the mantle layer (not shown). Also, the intensity of *Foxg1* staining seen in sections of all CHIR-treated slices was similar to that seen in DMSO-treated slices (Fig. 4f–h). Lef1 expression was as described earlier for each of the concentrations used. In both DMSO and CHIR-treated slices, Lef1 was detected in the nuclei of some cells in the VZ and the mantle layer of the ventral telencephalon. In these cells, the Lef1-positive nuclei (brown stain in Fig. 4f–h) were surrounded by cytoplasmic *Foxg1* mRNA (purple stain in Fig. 4f–h).

In both control and experimental groups, Ascl1-positive cells were detected in the VZ and mantle layer of the ventral telencephalon at all rostrocaudal levels (Fig. 4i–l). Ascl1 expression extended medially from the subpallial side of the PSPB and included the entire mediolateral extent in both zones. However, the number of cells expressing Ascl1 differed from one group to the other (Table 3). A mean of 215 (±SD = 44.71) Ascl1-positive cells per section was counted in the ventral telencephalon of DMSO-treated slices. In the VZ, a few of these Ascl1-positive cells were also Lef1-positive, but the majority were single-labelled (Fig. 4i^2^). Ascl1-positive cells in the mantle layer were all single-labelled. In 5 μM CHIR-treated slices, Ascl1 expressing cells present in the ventral telencephalon were ~3 % fewer than those counted in DMSO-treated slices and this difference was not statistically significant (Table 3). Many of the Ascl1-expressing cells detected were single-labelled (Fig. 4j^2^). In 25 and 50 μM CHIR-treated slices, Ascl1-positive cells were significantly reduced by ~46 % (*P* < 0.001) and 62 % (*P* < 0.001), respectively, compared to the number found in the control group (Table 3). The number of Lef1;Ascl1 double-labelled cells was not different between control and DMSO-treated slices (Table 4).

**Table 3:** Ascl1-positive (+) cells in the ventral telencephalon of treated slices

Ascl1-positive cells per section				
	Mean	STDEV	% Ascl1-+	*P* value
DMSO	215.40	44.71	100.00	N/A
5 μM CHIR	209.60	28.76	97.31	>0.05
25 μM CHIR	115.60	12.35	53.67	<0.001
50 μM CHIR	82.27	11.63	38.19	<0.001

Shown are the mean number of cells per section with the standard deviation (STDEV), the  % number of Ascl1-positive cells and the *P* values of each group compared to the control (DMSO) group

**Table 4:** Lef1 and Ascl1 (Lef1;Ascl1) double-positive cells in the ventral telencephalon of treated slices

Lef1;Ascl1-positive cells per section			
	Mean	STDEV	*P* value
DMSO	12.33	3.31	N/A
5 μM CHIR	13.83	4.37	>0.05
25 μM CHIR	14.08	4.08	>0.05
50 μM CHIR	14.25	5.10	>0.05

Shown are the mean number of cells per section with the standard deviation (STDEV) and the *P* values of each group. No significant differences are observed

These results confirm that the ventral telencephalon is competent to respond to Wnt/β-catenin signalling as shown by the increased expression of Lef1. In addition, this Lef1 upregulation leads to a decrease in the number of Ascl1-positive cells that are normally expressed in the ventral telencephalon.

### The EmT induces cell fate changes in the ventral telencephalon

To test whether signals from the EmT are capable of inducing cell fate changes similar to those observed when Wnt/β-catenin signalling is upregulated in the ventral telencephalon, EmT tissue from tau-GFP transgenic mice (donor) that ubiquitously express GFP (Pratt et al. 2000) and, therefore, allowed a distinction between donor and host tissue was transplanted into the ventral telencephalon of wild type CD1 mice (host) in culture. The hosts were then analysed for expression of Lef1, Foxg1 and Ascl1 in the ventral telencephalon.

**Fig. 5:**
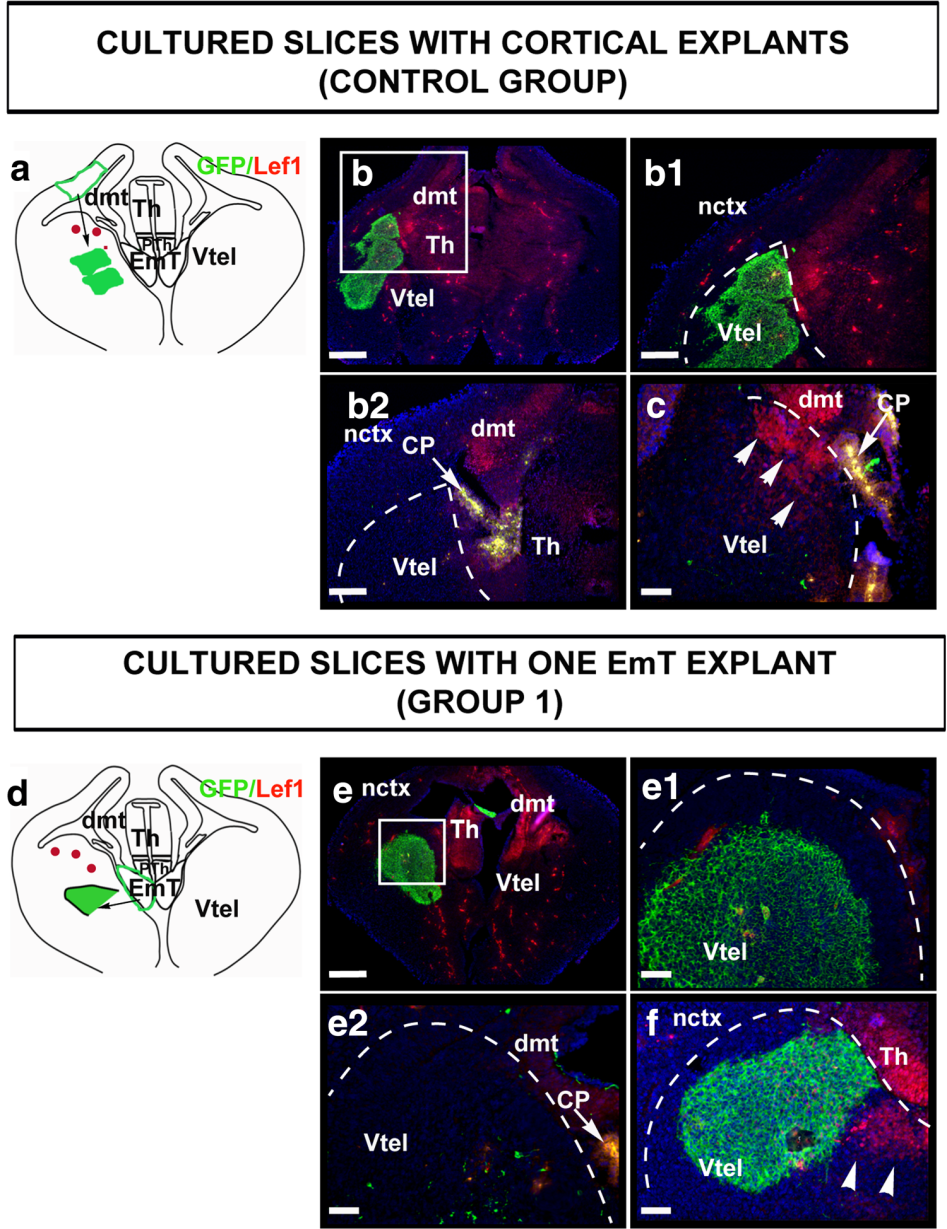
Lef1 expression in the ventral telencephalon of cultured slices hosting two cortical explants (**a**–**c**) and one EmT explant (**d**–**f**). **a** A schematic representation of Lef1 expression in slices hosting two cortical explants. The region of the tau-GFP donor tissue from which cortical explants were taken from is shown in green. In 9/10 of slices analysed, Lef1 staining was not seen in the ventral telencephalon. In the remaining 1/10 slices, Lef1 staining was seen in the ventricular zone but not in the mantle layer. **b**, **c** Sections of two slices from this group with two tau-GFP-positive cortical explants each on the left hemisphere. b^1^ is a higher magnification of b and b^2^ is a section from the same slice. Superficial is to the left. In b^1^ and b^2^, Lef1 staining is not seen in the ventral telencephalon. In c, Lef1-positive cells (*arrowheads*) are seen in the ventricular zone but not in the mantle layer. **d** A schematic representation of Lef1 expression in slices hosting one EmT explant from a tau-GFP donor is shown in *green*. In 9/10 of slices analysed, Lef1 was not detected in the ventral telencephalon. However, in the remaining slice Lef1 staining was seen in the ventricular zone of the ventral telencephalon, but not in the mantle layer. **e**, **f** Sections from cultured slices hosting one tau-GFP-positive EmT explant (*green*) on the left half of the brain. e^1^ and e^2^ are serial sections of e. Lef1 staining is not detected in these sections. In e^2^, very little of the explant remains. In f, Lef1 staining is seen in the ventricular zone around the explant (*arrowheads*). The *dashed curves* delineate the limits between the ventral telencephalon and the thalamus and/or dorsal telencephalon. The arrows in b^2^, c and e^2^ mark the CP, which shows intense *yellow* non-specific staining. Abbreviations: *CP* choroid plexus, *EmT* Eminentia thalami, *dmt* dorsomedial telencephalon, *nctx* neocortex, *PTh* prethalamus, *Th* thalamus, *Vtel* ventral telencephalon. *Scale bars* b = 250 μm, b^1^, b^2^, = 100 μm; c, e^1^, e^2^, f = 50 μm

**Fig. 6:**
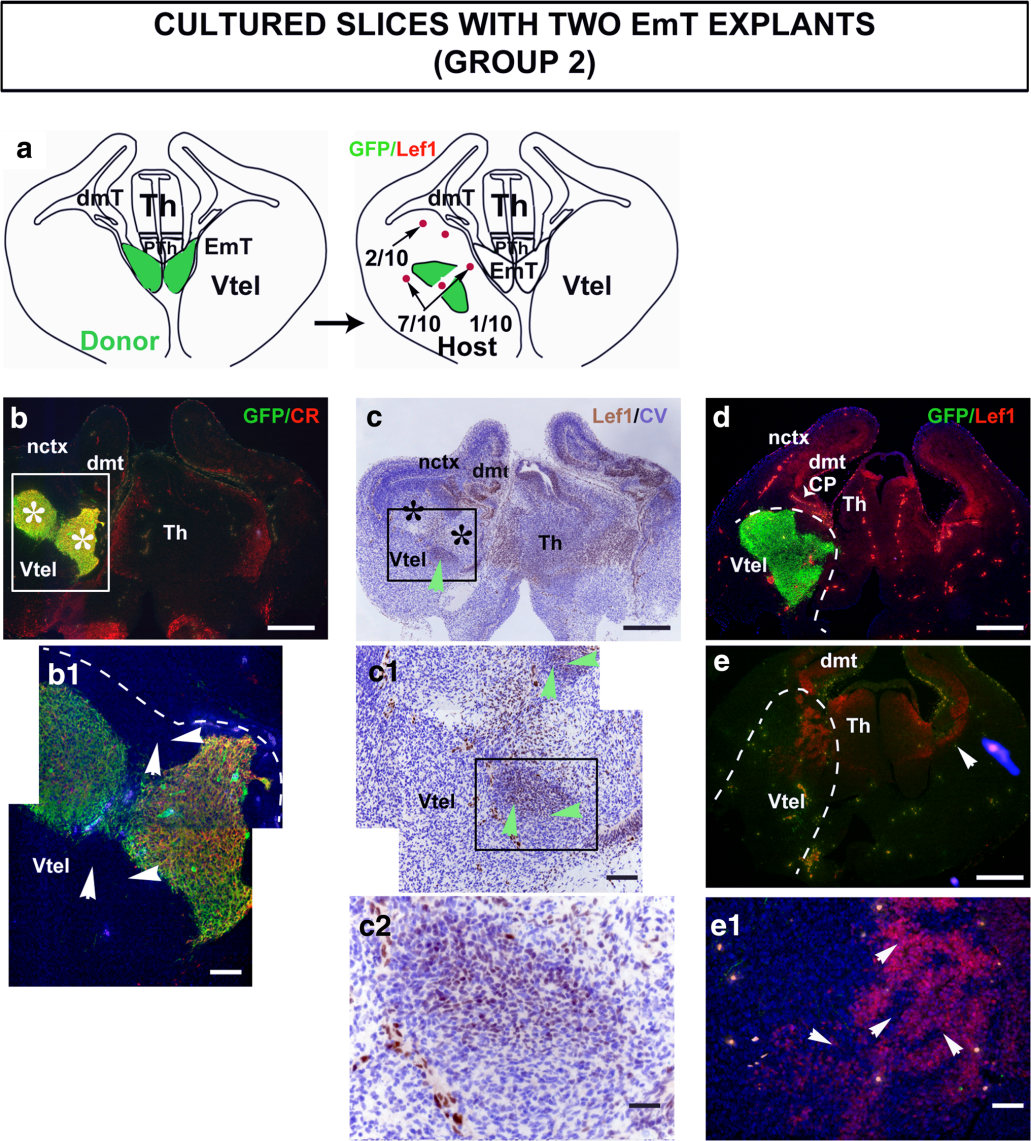
Lef1 expression in the ventral telencephalon of cultured slices hosting two EmT explants. **a** A schematic representation of Lef1 expression in this group. The region of the tau-GFP donor tissue from which the two EmTs were taken from is shown in *green*. In 1/10 of slices analysed, Lef1 was not detected in the ventral telencephalon; in 2/10 slices, Lef1 was seen only in the ventricular zone (VZ); and in 7/10 slices, Lef1 was seen in the VZ and mantle layer. (**b**) Double immunofluorescence for GFP (*green*) and Calretinin (CR) (*red*) on a section from a cultured slice hosting two EmT explants (a*sterisk*) on the left half of the brain. **b** ^**1**^ is a magnification of the region indicated in b. The explants are both GFP and Calretinin positive confirming that these are EmT tissue. **c** Lef1 immunohistochemistry (*brown* staining) counterstained with cresyl violet (*blue* staining) on a consecutive section to that shown in b reveals staining in cells peripheral to the explants (*green arrowhead*). These Lef1-positive cells surrounding the explants are not GFP-positive as inferred from their positions in b^1^ (*white arrowheads*) and c^1^ (*green arrowheads*). **b** ^**1**^ and c^**1**^ are magnification of the regions indicated in b and c, respectively. **c** ^**2**^ is a magnification of the region indicated in **c** ^**1**^. **d** and **e** are serial sections from a slice hosting two EmT explants on one half of the ventral telencephalon. In e, where there is no EmT explant remaining, Lef1 staining is found in the VZ and mantle layer. The arrowhead points to Lef1 expression limited at the ventricular zone of the untreated hemisphere. **e** ^**1**^ is a magnification of the Vtel shown in e. Lef1-positive cells found in the ventral telencephalon (*arrowheads*) are not GFP-positive suggesting that they may be host cells. The section is counterstained with DAPI (*blue*). The *dashed curves* delineate the limits between the ventral telencephalon and the thalamus and/or dorsal telencephalon. Abbreviations: *CP* choroid plexus; *dmt* dorsomedial telencephalon, *EmT* eminentia thalami; *nctx* neocortex; *PTh* prethalamus, *Th* thalamus, *Vtel* ventral telencephalon. *Scale bars* b, c, d, e = 250 μm; b^1^, c^1^, e^1^ = 50 μm; c^2^ = 20 μm

Two groups of EmT transplants were used. One group consisted of 10 slices, each hosting one piece of EmT explant in the ventral telencephalon (Fig. 5d, Group 1). The other group was made up of 10 slices with two EmT explants each (Fig. 6a, Group 2). To control for the specificity of the effects of the EmT tissue in the ventral telencephalon, an additional group of 10 slices each hosting two pieces of cortical explants from the middle third of the dorsolateral telencephalon, where Lef1 expression is very weak, was included in the study (Fig. 5a, Control group). In all three groups, the explants were placed in one hemisphere, and the opposite hemisphere was used as control. To confirm the location of the explants in the hosts, the slices were examined for GFP expression (green staining in Figs. 5, 6). Additionally in cultures hosting EmT explants, Calretinin immunofluorescence was done to confirm that they were indeed EmT tissue (Fig. 6b, b^1^ and not shown).

Double immunofluorescence for GFP (green) and Lef1 (red) revealed that in the control group, Lef1 was not detected in the ventral telencephalon of 9/10 slices (Fig. 5b, b^1^, b^2^). In 1/10 of slices analysed (Fig. 5c), Lef1-positive cells were seen in parts of the VZ, though no positive cells were detected in the mantle layer.

Similarly, in cultured slices hosting one EmT explant, Lef1 was not detected in the ventral telencephalon of 9/10 of the slices (Fig. 5e, e^1^, e^2^). In only 1/10 of slices analysed, the Lef1-immunoreactive cells were present in the VZ but not in the mantle layer, as indicated by arrowheads in Fig. 5f.

It should be noted that in some cases Lef1 expression was also observed in the VZ of the ventral telencephalon of the control hemisphere with no explant present (arrowhead in Fig. 6e). This is in line with Lef1 expression detected in a group of slices cultured without explant. In 4/20 of these control slices, Lef1 was detected in the VZ of the ventral telencephalon, either on one or both halves of the brain, but it was never observed in the mantle layer (not shown).

In cultured slices hosting two EmT explants, Lef1 staining was detected in the ventral telencephalon in 9/10 of slices analysed. In 2 of these 9 slices, the Lef1-positive cells were only found at the VZ and were not observed in the mantle layer (not shown). In 7 of the 9 slices, the Lef1-positive cells were present in the VZ, as well as the mantle layer (Fig. 6c, c^1^, c^2^, e, e^1^). Lef1-positive cells were found in sections where the explants were present, as revealed by their GFP immunoreactivity (compare position of explant in Fig. 6b, c), and were located around the explants (Fig. 6c, c^1^, c^2^). These Lef1-positive cells were GFP-negative (Lef1 brown staining in Fig. 6c^1^ and c^2^ is found outside the explant, the GFP-positive area shown in Fig. 6b^1^). Lef1-positive cells were also found in the VZ and the mantle layer of the ventral telencephalon of sections where no more tissue from the explant could be detected with GFP immunostaining and as before, these Lef1-positive cells were GFP-negative (Fig. 6e, e^1^). The fact that Lef1-expressing cells present in the mantle layer of the ventral telencephalon were GFP-negative indicates that these cells are unlikely to be donor cells and may be ventral telencephalic cells induced to express Lef1.

**Fig. 7:**
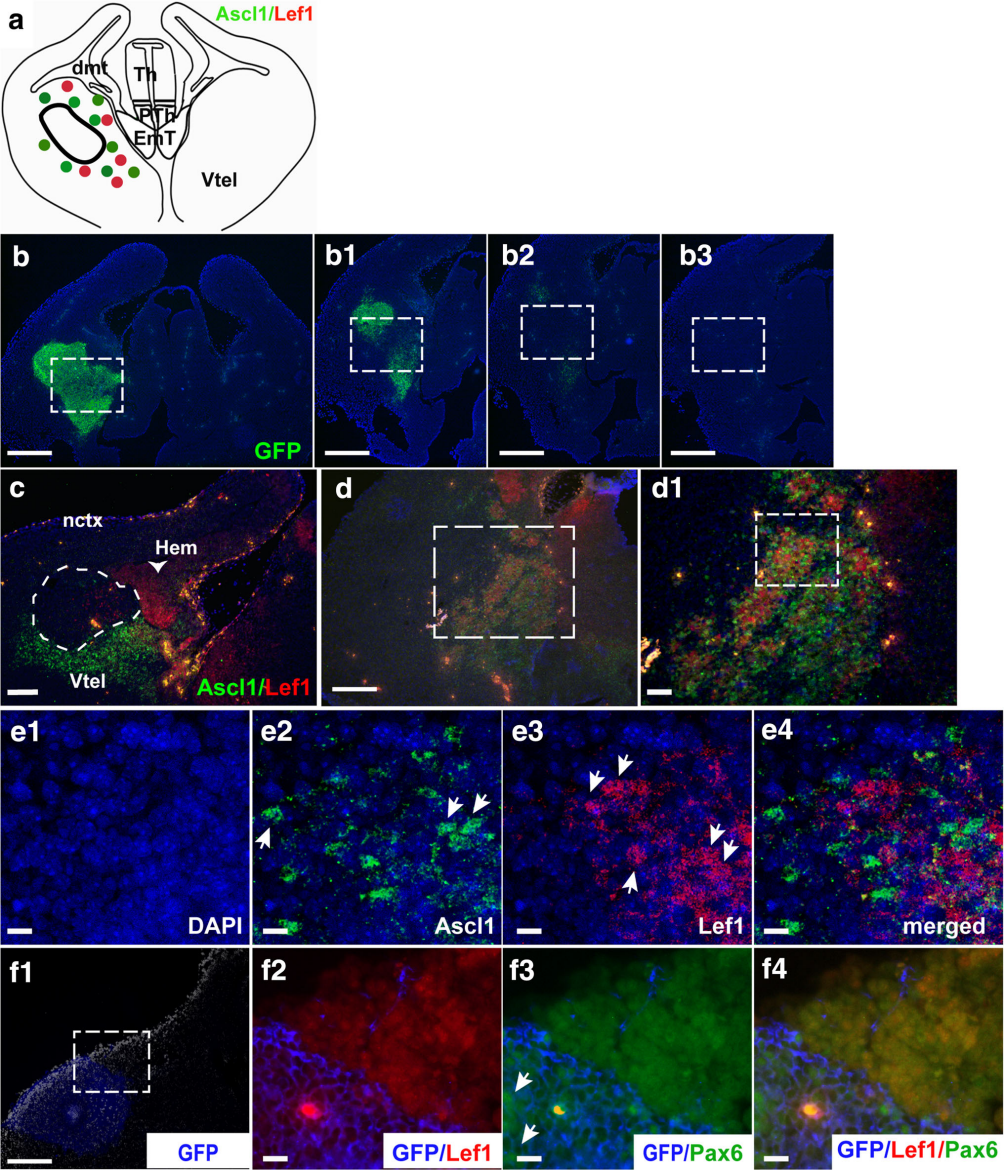
Ascl1, Lef1 and Pax6 expression in the ventral telencephalon of cultured slices. **a** A schematic representation of Ascl1 (*green dots*) and Lef1 (*red dots*) expression observed in slices with EmT explants. In sections with explants present, Ascl1 was not seen within the explants (depicted with a *black outline*) but was found in ventral telencephalic cells surrounding them. In slices hosting two EmT explants, Lef1-positive cells mingled with Ascl1-positive cells within the ventral telencephalon and outside the explant region. **b**–**b** ^**3**^ Serial cryostat sections of a slice culture with two tau-GFP EmT explants on the left hemisphere. GFP immunofluorescence shows explant position in **b**. Sections (**b** ^**1**^–**b** ^**3**^) are serial sections located in deeper layers to **b**. In these sections, the GFP staining is gradually reduced (**b** ^**1**^ **, b** ^**2**^) and eventually disappears **(b** ^**3**^ **)**. **c** A section with an EmT explant present (*outlined in white*). Ascl1 staining is seen in the ventricular zone and mantle layer around the explant but not in the explant per se. **d**, **d** ^**1**^ Ascl1 and Lef1 expression in the experimental half of a slice hosting two EmT explants. Both Ascl1-positive (*green*) and Lef1-positive (*red*) cells are seen in the ventricular zone and mantle layer of the ventral telencephalon. The *dotted square* in d outlines the high power image in d^1^. d is a section adjacent to b^3^. As with b^3^, the explant is not visible anymore in d. However, its position corresponds to that of the *dotted squares* in *panels* b–b^3^. At higher magnifications (**e** ^**1**^–**e** ^**4**^), many single-labelled Lef1-positive cells (*arrows* in e^3^) are found mingling with single-labelled Ascl1-expressing cells (*arrows* in e^2^). *Blue staining* (b–b^3^; e^1^–e^4^) is DAPI counterstain. **f** ^**1**^–**f** ^**4**^ Lef1 and Pax6 expression in the experimental half of a host slice. The *blue staining* in **f** ^**1**^ corresponds to the tau-GFP EmT explant. Dorsal to the explant, a group of Lef1-positive cells are found (*red* in f^**2**^). Most of these cells seem to co-express the dorsal telencephalic marker Pax6 (*green* in **f** ^**3**^, **f** ^**4**^). The *dotted square* in f^1^ corresponds to the high magnifications in f^2^–f^3^. The *arrows* in f^3^ point to Pax6-positive cells within the GFP-positive EmT. Abbreviations: *dmt* dorsomedial telencephalon; *EmT* eminentia thalami; *nctx* neocortex; *PTh* prethalamus, *Th* thalamus, *Vtel* ventral telencephalon. *Scale bars* b–b^3^, f^1^ = 200 μm; c, d = 100 μm; d^1^, f^2^–f^4^ = 50 μm; e^1^–e^4^ = 25 μm

To gain further insight into the identity of these Lef1-expressing cells, we examined whether they expressed the ventral telencephalic marker Ascl1. In sections containing EmT explants, Ascl1 staining was not seen in the explants but was found in cells surrounding them, as shown in Fig. 7c and illustrated in Fig. 7a. Normal expression of Ascl1 and Lef1 was observed in the cultured half of the brain with no explant (not shown). In sections with explants containing Lef1-induced cells, the Lef1-positive cells mingled with the Ascl1-positive cells (Fig. 7d, d^1^ and illustrated in Fig. 7a). Figure 7d and d^1^ depicts sections of the slice culture located deep within the explant. The position of the explant for those sections is shown in Fig. 7b–b^3^. High power confocal images for these slice explants immunoreacted with Ascl1 and Lef1 showed that most of these cells either expressed Lef1 or Ascl1 (Fig. 7e^1^–e^4^). Further analysis of the identity of the Lef1-positive cells using in situ hybridization for *Foxg1* revealed that the Lef1-expressing cells in the ventral telencephalon also expressed *Foxg1* (not shown).

We further examined whether the Lef1-positive cells induced in the ventral telencephalic tissue surrounding the explant expressed the dorsal telencephalic marker Pax6. Lef1-positive cells intermingling with Ascl1-positive cells located lateral to the explant in Fig. 7f^1^ were immunonegative for Pax6 (not shown). However, a group of Lef1-positive cells located dorsal to this explant (Fig. 7f^1^, f^2^), in a region that does not normally express Pax6, were also positive for Pax6 (Fig. 7f^3^, f^4^). Interestingly, at this location which would not normally express Ascl1 either, no Ascl1-positive cells were observed (not shown). This suggests that the molecular fates of cells affected by the explant might depend on their location and pre-existing molecular state.

All together, the above results indicate that EmT tissue has the capacity to activate Wnt/β-catenin signalling in the ventral telencephalon, a brain region where it is not normally active, and to suppress expression of the ventral telencephalic marker Ascl1. Abnormal expression of Pax6 in the ventral telencephalon suggests that the presence of EmT may, in certain circumstances, cause the ventral telencephalon to adopt a dorsal fate.

## Discussion

The EmT is a diencephalic structure that lies between the choroid plexus of the lateral ventricle and the prethalamus (Puelles and Rubenstein 2003 and Fig. 1). Although its location has been carefully mapped in vertebrates, including humans, using anatomical landmarks and gene expression patterns (Abellan et al. 2010; Fotaki et al. 2006, 2008; Puelles et al. 2000; Retaux et al. 1999; Roy et al. 2014), little is known about its function. In contrast to non-mammalian EmT, which has been identified both during development and in adults (Wullimann and Mueller 2004), the mammalian EmT seems to be a transient developmental structure (Keyser 1972). Based on its transient appearance, characterized by strong calretinin immunoreactivity which becomes undetectable after E17.5, Abbott and Jacobowitz (1999) suggested that the EmT may act as diencephalic organizing centre. A recent study suggested that the cortical hem, ventral telencephalic septum, and EmT may act as a multicomponent patterning and organizing centre for the medial forebrain (Roy et al. 2014). Here, we examine the molecular and cellular properties of the EmT and provide strong evidence that is in line with the notion that this transient developmental mammalian structure may act as a signalling centre of the developing forebrain.

Our detailed analysis of expression of members of the *Wnt*, *Bmp* and *Fgf* gene families in the developing EmT revealed that, indeed, this structure is rich in expression of signalling molecules. For most of these molecules, expression was detected in the proliferative layer of the EmT at around E11.5 and ceased by E15.5, when most of the progenitor cells have differentiated into neurons, as reflected by the thinning of the size of the ventricular zone and the thickening of Calretinin staining. Unlike the neighbouring cortical hem, a secondary organizer of the hippocampal primordium (Mangale et al. 2008), which displays strong expression for both *Wnt* and *Bmp* molecules (Grove et al. 1998), the EmT showed strong expression for *Wnts* but low levels of expression for members of the *Bmp* family. *Fgfs* showed restricted expression in the EmT but expression of *Mkp3*, a downstream negative regulator of Fgf signalling (Li et al. 2007), was more widespread. This compares to the restricted expression of *Fgf8* in relation to the widespread expression domain of *Mkp3* in the isthmus (Echevarria et al. 2005), a secondary organizer of the midbrain–hindbrain region (Martinez 2001).

In addition to strong expression of *Wnt* molecules, mainly *Wnt7b* and *Wnt8b*, the EmT showed expression of downstream targets of the Wnt/β-catenin pathway such as *Axin2* and *Lef1*. *Axin2*, although strong, was only found in the most lateral aspect of the EmT in its entire rostrocaudal axis. This restricted expression may be due to the strong expression of *Sfrp2*, an antagonist of Wnt signalling (Wawrzak et al. 2007), expressed in a high-medial to low-lateral gradient complementary to that of *Axin2*. Very low *Lef1* expression in the EmT with high levels in the neighbouring prethalamus resembles lack of *Lef1* expression in the cortical hem with high expression levels in the adjacent dorsomedial pallium (Fotaki et al. 2011; Galceran et al. 2000).

Can the secreted proteins found to be expressed in the EmT elicit a signalling response? The fact that our heterotopic explants of EmT donor tissue within the ventral telencephalic host led to upregulation of Lef1 in the surrounding ventral telencephalon in 9/10 of slices examined favours our hypothesis that signals emitted from the EmT may affect the fate of surrounding cells. Although very faintly Lef1-expressing cells were detected in the VZ of the ventral telencephalon in wild type embryos, none were found in the mantle layer. In contrast, the Lef1-expressing cells around the explant were strongly expressing cells and in 7/10 cases they were not restricted to the VZ, but were also found in the mantle layer, strongly indicating that the grafted EmT induces production of these cells. The use of tau-GFP mice that express ubiquitously GFP (Pratt et al. 2000) allowed us to unequivocally distinguish donor from host tissue. Although the EmT has been previously shown to be a source of neurons that migrate to the ventral forebrain (Tissir et al. 2009), in our case the Lef1-positive cells were GFP-negative, indicating that it is unlikely that these cells are EmT-migrating cells. As we only observed Lef1 upregulation in the VZ in 1/10 of control cases that involved transplanted cortical tissue in the ventral telencephalon, we are confident that our results are specific to EmT activity on the host environment rather than the outcome of a stochastic event. It is noteworthy that the effect of the EmT on the host tissue was only observed when two EmT pieces were grafted. It may be that although the EmT possesses an apparent ability to induce Lef1 in the ventral telencephalon ex vivo, under our experimental conditions the level of signal(s) secreted by one piece of EmT tissue does not reach the required threshold for this induction to take place.

It is also possible that the effect exerted on the surrounding tissue depends on the position of the explant. In the current experimental design, the explants were placed away from the VZ but placing them nearer to it might have led the EmT donor-emitted Wnt signals to cause a more profound effect on the surrounding tissue than the one we have currently observed. This is in line with the fact that in our CHIR-induced cultures we observed a clearer upregulation of Lef1-positive cells in the VZ rather than the mantle region.

Based on the fact that among the signalling factors we studied, *Wnt7b* and *Wnt8b* displayed the most robust and prolonged expression, we hypothesized that the primary pathway activated by the EmT is the Wnt/β-catenin signalling cascade. In cultured ventral telencephalic slices, where Wnt/β-catenin signalling was activated by inhibition of Gsk3β activity (van Noort et al. 2002), Lef1 expression increased with an increased inhibition of this activity. The high levels of Lef1 expression in DMSO-treated slices have been previously observed in other culture systems suggesting that DMSO per se may activate Wnt/β-catenin signalling (Nakamura et al. 2003; Peng et al. 2002). However, these were significantly lower than the number of Lef1-expressing cells observed with even the lowest concentration of drug activator, confirming that the significant increase in Lef1-expressing cells in the ventral telencephalon was the result of a specific activation of the Wnt/β-catenin pathway. Although some of the Lef1-positive cells co-expressed Ascl1, the number of these Lef1;Ascl1-positive cells was very small and remained unchanged among all treated conditions and the control group, suggesting that these double-labelled cells may account for the low number of Lef1-positive cells normally found in the ventral telencephalon.

The dose-dependent induction of Lef1 in these ventral slices was concomitant with a significant dose-dependent decrease in Ascl1 expression, strongly suggesting that Lef1 upregulation in the ventral telencephalon leads to a suppression of ventral fates. Similarly, when the induced Lef1-positive cells in our explant experiment were examined for expression of the ventral telencephalic marker Ascl1 and the telencephalic marker *Foxg1*, it was found that these cells were Ascl1-negative;Foxg1-positive. Thus, results of these two different experimental approaches suggest that Wnt/β-catenin signalling may lead to a cell fate change in the surrounding tissue. It is worth noting that some of the Lef1-induced cells were in deeper positions, relative to the VZ apical surface, than those that showed induction in the CHIR-treated explants. One likely explanation for this is that the EmT grafts produce signalling molecules additional to Wnts such as Fgfs and Bmps, which may affect exactly which cells undergo Lef1 activation in the host tissue.

If the EmT-derived signals inhibit Ascl1-expressing cells, why are there still a lot of Ascl1-positive cells surrounding the EmT explants? We propose that the EmT-secreted signals are able to affect the fate of some cells which respond to those signals by upregulating Lef1. One reason that some cells do not respond by upregulating Lef1 may be that their state of differentiation renders them unresponsive to the signal. Alternatively, the levels of signal reaching each cell may differ among cells due to their precise position and the nature of the diffusion of the signal from the EmT explant. In addition, as discussed above, the EmT expresses different secreted factors along its rostrocaudal and medio-lateral axis and their expression levels change during embryonic development. In line with this dynamic spatio-temporal expression, EmT cells may elicit a different response at different positions in the surrounding tissue due to spatial variation in the cocktail of molecules produced by the explants and/or in the relative amounts of these secreted factors.

In agreement with the role of Wnt/β-catenin signalling in dorsal telencephalic specification (Backman et al. 2005; Gunhaga et al. 2003) and the fact that constitutive expression of β-catenin in the mouse ventral telencephalon leads to its partial dorsalization with a concomitant repression of ventral telencephalic markers (Backman et al. 2005), it is possible that these Lef1-positive cells have acquired dorsal telencephalic identity. This is further supported by the upregulated expression of the dorsal telencephalic marker Pax6 within the ventral telencephalon adjacent to the EmT explant. It is intriguing that this Pax6 expression was observed in cells ectopically expressing Lef1 that were not surrounded by Ascl1 expressing cells but was absent from Lef1-positive cells surrounded by Ascl1-positive cells. Further studies with additional markers will be needed to test further the identities of the EmT-induced Lef1-positive cells within the ventral telencephalon and to allow us to understand the necessary conditions that trigger those identities.

A self-evident question that this study raises is that if the EmT plays a role as signalling centre, which are the domains it exerts its signalling activity on? Signalling centres usually lie at the borders of the regions they specify (Kiecker and Lumsden 2005). As the EmT lies between the choroid plexus of the lateral ventricle, the ventral telencephalon and the prethalamus, an attractive hypothesis is that it may pattern these regions (Online Resources 1, 2 &3). The phenotypic defects observed in mouse mutants with abnormal EmT structures favour this hypothesis. In the *Gli3* mutant, EmT cell clusters intermingle with dorsal telencephalic cells possibly as a result of abnormal formation of the telencephalic-diencephalic boundary (Fotaki et al. 2006). In addition, in *Gli3* chimaeras the EmT seems to become duplicated near the telencephalic–diencephalic border, where nearby cells express abnormal sets of transcription factors—cells lying on the telencephalic side of the boundary express diencephalic markers (Quinn et al. 2009). In the *Lhx2* mutant, the EmT expands at the expense of the caudal ganglionic eminence, leading to the hypothesis that the EmT may act as the diencephalic hem to regulate key aspects of forebrain patterning and development (Roy et al. 2014). In the *Olig2* mutant, the EmT is expanded at the expense of a hypoplastic prethalamus (Ono et al. 2014). In all the above examples, changes in the mutant EmT are concomitant with significant changes in the above-mentioned neighbouring forebrain tissue.

Our findings show that the EmT not only expresses Wnt molecules but also activates Wnt/β-catenin signalling. At present, we can only hypothesize about the forebrain areas to which the EmT Wnts may be signalling. Based on the fact that β-catenin promotes and maintains thalamic fate but prevents prethalamic fate (Bluske et al. 2012), it is unlikely that EmT-derived Wnt/β-catenin signals target the neighbouring prethalamus. An attractive hypothesis is that, similar to the Wnt-rich cortical hem which has been proposed to affect choroid plexus development (Grove et al. 1998), Wnt signals from the EmT also affect formation and/or function of the adjacent choroid plexus. This is in line with the fact that already at 8 somites, before choroid plexus formation, a *Wnt7b*/*Wnt8b* rich domain defines a region of the prosencephalon that probably corresponds to the future EmT and adjacent cortical hem (Fotaki et al. 2011).

A comprehensive fate map of the mammalian EmT has not been reported to date. It has been shown that pioneer neurons from the mouse EmT migrate to the ganglionic eminences and the marginal zone of the cortex (Morante-Oria et al. 2003) and also form part of the ventral forebrain (Tissir et al. 2009). It has also been reported that the EmT gives rise to the bed nucleus of the stria medullaris and that of the posterior part of the stria terminalis (Puelles et al. 2000). Although our current study cannot provide any information on the fate of the EmT, adapting our experimental protocol using homotopic explants from tau-GFP mouse donors could generate valuable data regarding the fate of EmT cells.

## Conclusions

The EmT is a Wnt-rich expressing region with an ability to induce the fate of surrounding cells away from its original source. These properties strongly support its role as a forebrain signalling centre. Future experiments will explore further this potential and will also examine the properties of the other signalling molecules expressed in the EmT.

## Electronic supplementary material

Below is the link to the electronic supplementary material.

Online Resource 1. Optical Projection Tomography (OPT) image of an E12.5 wild type embryo head revealing the position of the EmT (green) and that of its surrounding structures. The EmT may exert its signalling effect, indicated by dotted arrows and question marks, to the ventral telencephalon (yellow area), the prethalamus (blue), the choroid plexus (orange) and/or the adjacent cortical hem (red). (TIFF 1830 kb)

Online Resource 2. A montage of all the OPT images that constitute the animation in the Online Resource 3. Only the forebrain structures that are adjacent to the EmT (depicted in green) are colour-coded. The ventral telencephalon is depicted in yellow only in sections that are in close proximity to the EmT; prethalamus in blue, choroid plexus in orange, cortical hem in red. (TIFF 23569 kb)

Online Resource 3. Three-dimensional depiction of the EmT and its surrounding structures in an E12.5 wild type embryo (see also Online Resource 2). (AVI 8529 kb)
