# Supplementary figures and images for: The molecular and cellular signatures of the mouse eminentia thalami support its role as a signalling centre in the developing forebrain
Source: Brain Struct Funct. 2015 Oct 12;221(7):3709–27. doi: 10.1007/s00429-015-1127-3 (PMC5009181; doi:10.1007/s00429-015-1127-3)

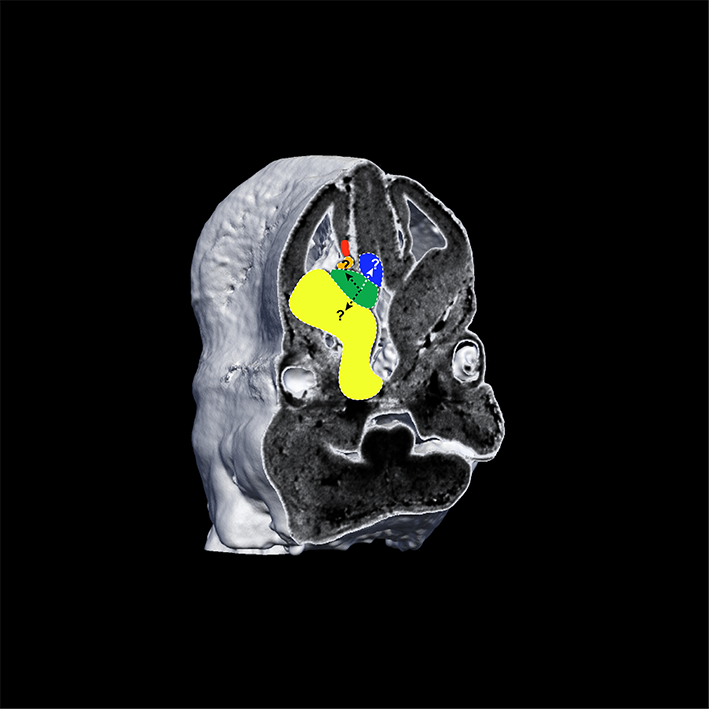

Supplement: Supplementary file 1 — Online Resource 1. Optical Projection Tomography (OPT) image of an E12.5 wild type embryo head revealing the position of the EmT (green) and that of its surrounding structures. The EmT may exert its signalling effect, indicated by dotted arrows and question marks, to the ventral telencephalon (yellow area), the prethalamus (blue), the choroid plexus (orange) and/or the adjacent cortical hem (red). (TIFF 1830 kb) [file 429_2015_1127_MOESM1_ESM.tif]

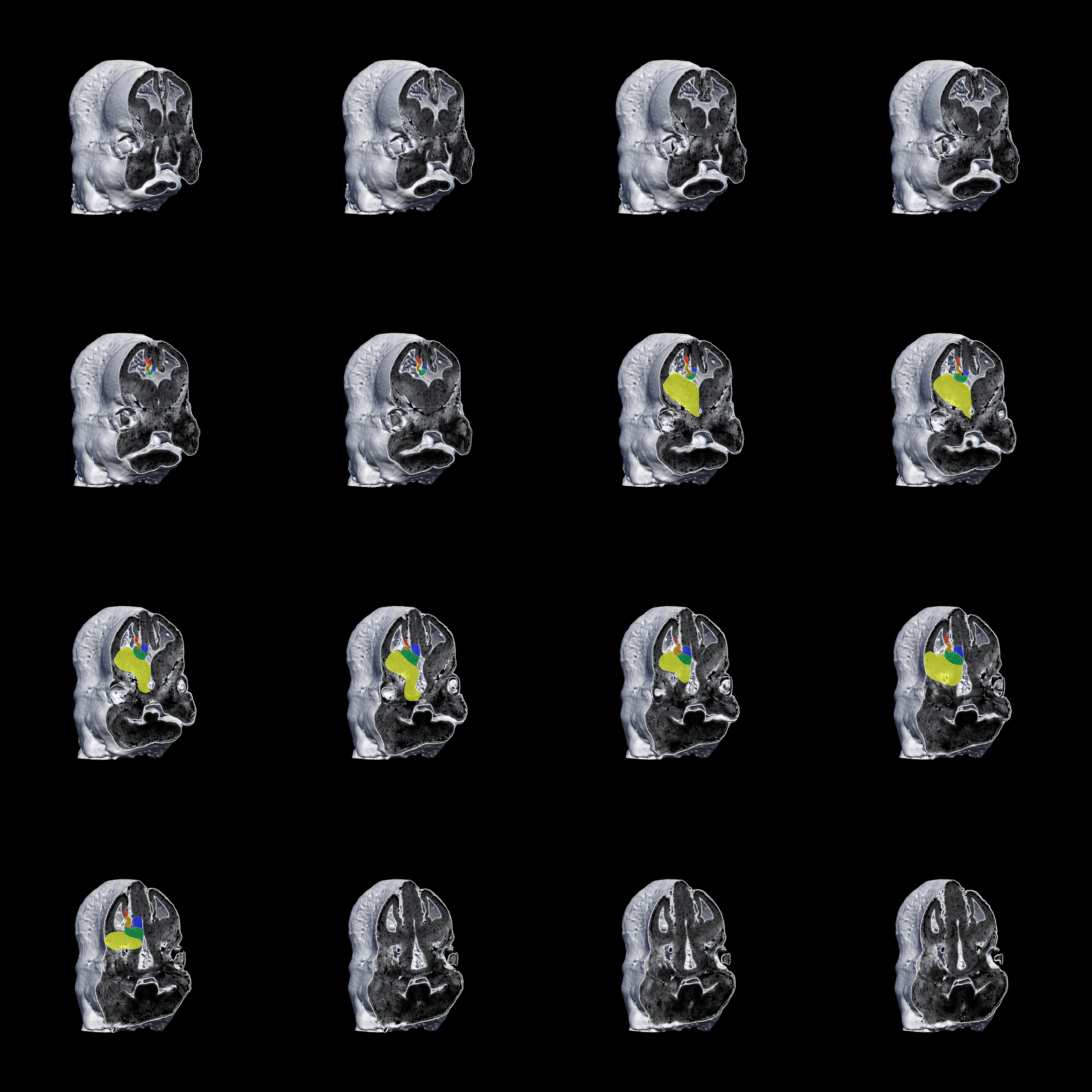

Supplement: Supplementary file 2 — Online Resource 2. A montage of all the OPT images that constitute the animation in the Online Resource 3. Only the forebrain structures that are adjacent to the EmT (depicted in green) are colour-coded. The ventral telencephalon is depicted in yellow only in sections that are in close proximity to the EmT; prethalamus in blue, choroid plexus in orange, cortical hem in red. (TIFF 23569 kb) [file 429_2015_1127_MOESM2_ESM.tif]
